# Performance of Hitchens-Pike-Todd-Hewitt Medium for Group B Streptococcus Screening in Pregnant Women

**DOI:** 10.1371/journal.pone.0123988

**Published:** 2015-04-16

**Authors:** Simone Cristina Castanho Sabaini de Melo, Angela Andréia França Gavena, Flávia Teixeira Ribeiro Silva, Ricardo Castanho Moreira, Regiane Bertin de Lima Scodro, Rosilene Fressatti Cardoso, Vera Lúcia Dias Siqueira, Rúbia Andreia Faleiros de Pádua, Maria Dalva de Barros Carvalho, Sandra Marisa Pelloso

**Affiliations:** 1 Postgraduate Program in Health Sciences, State University of Maringá, Maringá, Paraná, Brazil; 2 Nursing Sector, State University of Northern Paraná, Bandeirantes, Paraná, Brazil; 3 Department of Clinical Analysis and Biomedicine, State University of Maringá, Maringá, Paraná, Brazil; 4 Department of Medicine, State University of Maringá, Maringá, Paraná, Brazil; 5 Department of Nursing, State University of Maringá, Maringá, Paraná, Brazil; Columbia University, UNITED STATES

## Abstract

Group B streptococcus (GBS), which commonly colonizes the female genital tract and rectum, can cause infections in newborns with varying severity, possibly leading to death. The aim of the present study was to evaluate Hitchens-Pike-Todd-Hewitt (HPTH) medium performance for GBS screening in pregnant women. A descriptive analytical cross-sectional study was performed with 556 pregnant women, of which 496 were at 35-37 weeks of gestation and 60 were at ≥ 38 weeks of gestation. The study was conducted from September 2011 to March 2014 in northern Paraná, Brazil. Vaginal and anorectal clinical specimens from each pregnant woman were plated on sheep blood agar (SBA) and seeded on HPTH medium and Todd-Hewitt enrichment broth. Of the 496 pregnant women at 35-37 weeks of gestation, 141 (28.4%) were positive for GBS, based on the combination of the three culture media and clinical specimens. The GBS colonization rates that were detected by each medium were 22.2% for HPTH medium, 21.2% for SBA, and 13.1% for Todd-Hewitt enrichment broth. Of the 60 pregnant women at ≥ 38 weeks of gestation, seven (11.7%) were positive for GBS. These results demonstrate that HPTH medium and SBA were more sensitive than Todd-Hewitt enrichment broth for GBS screening in pregnant women and good GBS recovery in culture, indicating that the two media should be used together for vaginal and anorectal specimens.

## Introduction

Group B streptococcus (GBS) or *Streptococcus agalactiae* is a Gram-positive cocci that commonly colonizes the female genital tract and rectum [1]. GBS can cause infections in women and men, presenting as cystitis, skin infections, among others. There can be major implications for pregnant women that cause impairments in pregnancy, chorioamnionitis, abortion, intrauterine fetal death, premature membrane rupture, preterm delivery, postpartum endometritis, and sepsis [2].

The prevalence of GBS colonization in pregnant women worldwide has been reported to vary from 3% to 45% (3). In Brazil, the GBS colonization rates feature variations according to demographic region. The rates reported by some author showed ranged from 5% to 30% [3–6].

An estimated 1–2% of newborns from colonized mothers develop some diseases caused by GBS, which can vary in severity and cause death [7]. In developing countries, such as Brazil, the incidence of neonatal GBS infection ranges from 0.80 to 3.06 per 1,000 live births [8].

GBS infection can be transmitted to the newborn during labor or in utero from the mother’s vaginal and anorectal colonized mucosa. Screening for GBS carriage is imperative in pregnant women. Recommendations have been made to screen for GBS at 35–37 weeks of gestation [9] because this time period correlates with the delivery timeframe.

The U.S. Centers for Disease Control and Prevention (CDC) recommends using selective enrichment medium for GBS screening, such as Todd-Hewitt broth supplemented with antibiotics, followed by subculture on sheep blood agar [9]. However, some studies have demonstrated success using other media to detect GBS in pregnant women [10–14].

Although Todd-Hewitt enrichment broth is widely used because it is recommended by the CDC for GBS screening in pregnant women, the sensitivity and specificity of such methods still need to be evaluated. A previous study in Brazil [12] compared the sensitivity of Hitchens-Pike-Todd-Hewitt (HPTH) medium, Todd-Hewitt enrichment broth, and blood agar direct plating and found that HPTH medium had the best performance for GBS screening in anorectal and vaginal samples. However, the above study was performed with a small number of pregnant women, and GBS colonization rates are unknown in southern Brazil. Therefore, the aim of the present study was to evaluate the performance of HPTH medium for GBS screening in pregnant women.

## Materials and Methods

### Study population

The study was approved by the Ethics and Human Research Committee, State University of Maringa (no. 236/2011). The term of consent was read and signed by all study participants. A descriptive analytical cross-sectional study was performed with 556 pregnant women, of which 496 were at 35–37 weeks of gestation and 60 were at ≥ 38 weeks of gestation. The study was conducted from September 2011 to March 2014 in 21 municipalities in northern Paraná, Brazil. The gestational age was determined based on the last menstrual period. When the last menstrual period was indeterminate, we used fetal ultrasound in the first trimester of pregnancy. The exclusion criteria were previous use of antibiotics and/or vaginal cream in the last 7 days by the pregnant women.

### Clinical specimens and culture

Clinical specimens from the vaginal distal third were collected by introducing sterile swabs labeled 1, 2, and 3, without using a speculum. Anorectal specimens were collected by introducing sterile swabs labeled 4, 5, and 6 in the anorectal region.

Vaginal swab 1 and anorectal swab 4 specimens were immediately plated on 5% desfibrinated sheep blood agar-SBA (Himedia, Curitiba, Paraná, Brazil) and incubated at 35–37°C for 18–24 h.

Vaginal swab 2 and anorectal swab 5 were each submerged in 2ml of HPTH medium [12], and 100 μl of sterile defibrinated sheep blood (Laborclin, Pinhais, Paraná, Brazil) was added, followed by incubation at 35–37°C for 18–24 h. Afterward, subculturing was performed on SBA, with incubation at 35–37°C for 24–48 h.

Vaginal swab 3 and anorectal swab 6 were submerged in Todd-Hewitt medium (Himedia, Curitiba, Paraná, Brazil) supplemented with 8 μg/ml gentamicin (Inlab, São Paulo, Brazil) and 15 μg/ml nalidixic acid (Inlab, São Paulo, Brazil) according to the manufacturer's instructions and incubated at 35–37°C for 18–24 h. Afterward, an aliquot of each culture was plated on SBA (Himedia, Curitiba, Paraná, Brazil) and incubated at 35–37°C for 24–48 h.

### GBS biochemical and serological identification

The culture interpretation and identification of GBS were performed in the Laboratory of Clinical Bacteriology, Department of Clinical Analyses and Biomedicine, State University of Maringá, Paraná, Brazil. Colonies that were suggestive of GBS (beta- and non-hemolytic) were subjected to microscopy (Gram stain), biochemical identification (catalase, bile esculin, and hippurate hydrolysis), and latex agglutination using a streptococcal grouping kit (Oxoid, Hampshire, UK) according to the manufacturer's instructions.

### Data analysis

The data were analyzed by chi-square using Epidata software version 3.1. Values of p<0.05 were considered statistically significant.

## Results

**Fig 1:**
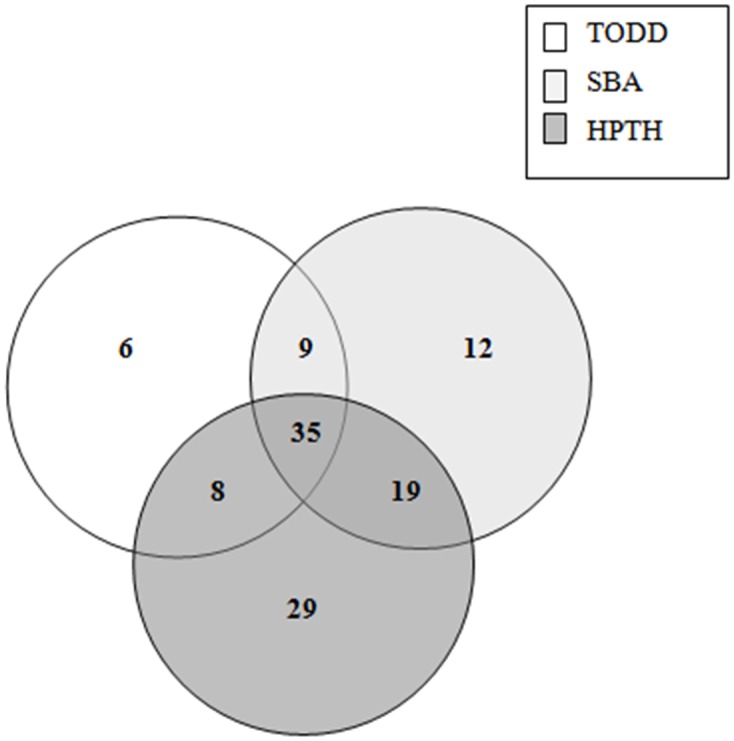
Number of *Streptococcus agalactiae* positive pregnant women (35–37 weeks of gestation) detected by HPTH medium, Todd-Hewitt enrichment broth (TODD), and sheep blood agar (SBA) plating in vaginal samples.

**Fig 2:**
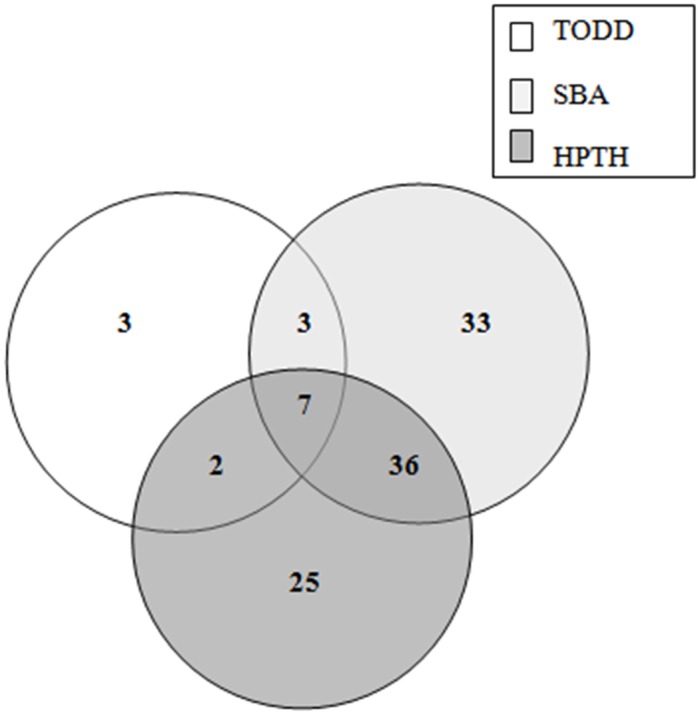
Number of *Streptococcus agalactiae* positive pregnant women (35–37 weeks of gestation) detected by HPTH medium, Todd-Hewitt enrichment broth (TODD), and sheep blood agar (SBA) plating in anorectal samples.

Of the 496 pregnant women at 35–37 weeks of gestation who participated in the study, 141 (28.4%) were positive for GBS based on the combination of the three culture media for the two clinical specimens (S1 Table). The GBS colonization rate detected was 22.2% for HPTH medium, 21.2% for SBA, and 13.1% for Todd-Hewitt enrichment broth (Table 1). Of the pregnant women who were positive for GBS, based on HPTH medium, 8.1% were positive exclusively in vaginal specimens, 3.8% were positive exclusively in anorectal specimens, and 10.3% were positive in both specimens. SBA plating revealed GBS-positive cultures in 5.2% of the vaginal specimens and 6.0% of the anorectal specimens, and 9.9% were positive in both specimens. Plating with Todd-Hewitt enrichment broth revealed GBS-positive cultures in 10.1% of the vaginal specimens and 1.4% of the anorectal specimens, and 1.6% were positive in both specimens (Figs 1 and 2, Tables 1 and 2).

**Table 1:** Prevalence of *Streptococcus agalactiae* in pregnant women at 35–37 weeks of gestation, based on clinical specimens tested with Todd-Hewitt enrichment broth (TODD), sheep blood agar (SBA), and Hitchens-Pike-Todd-Hewitt (HPTH) culture media.

Test	n	%	95% Confidence interval	Chi-square	*p* value
**Vaginal**				8.1473	0.0170
HPTH	40	8.1	5.66–10.46		
SBA	26	5.2	3.28–7.20		
TODD	50	10.1	7.43–12.73		
**Anorretal**				14.7231	0.0006
HPTH	19	3.8	2.14–5.52		
SBA	30	6.0	3.95–8.15		
TODD	7	1.4	0.37–2.45		
**Anorectal and vaginal**				35.2594	<0.0001
HPTH	51	10.3	7.61–12.95		
SBA	49	9.9	7.25–12.51		
TODD	8	1.6	0.50–2.72		

**Table 2:** Comparison of *Streptococcus agalactiae* detection by Todd-Hewitt enrichment broth (TODD), sheep blood agar (SBA), and Hitchens-Pike-Todd-Hewitt (HPTH) culture media in pregnant women at 35–37 weeks of gestation.

	n	95% Confidence interval	Chi-square	*p* value
**Media**			16.0464	0.0003
TODD	65/496	13.1 (10.13–16.07)		
SBA	105/496	21.2 (17.57–24.77)		
HPTH	110/496	22.2 (18.52–25.84)		
**Test post-hoc**				
TODD *versus* SBA	65 *versus* 105	13.1 *versus* 21.2	10.7974	0.0010
TODD *versus* HPTH	65 versus 110	13.1 *versus* 22.2	13.4325	0.0002
SBA *versus* HPTH	105 versus 110	21.2 *versus* 22.2	0.0950	0.7579

HPTH had higher performance in detecting GBS in vaginal specimens, which accounted for 91 (18.3%) pregnant women. For anorectal specimens, which include patients with positive culture only in anorectal and anorectal plus vaginal specimens, the best performance for GBS screening was observed with SBA direct plating (15.9%) (Figs 1 and 2, Tables 1 and 2).

Of the 60 pregnant women at ≥ 38 weeks of gestation, seven (11.7%) were positive for GBS. Of these, one was detected only by HPTH, one only by SBA plating, and five were detected by both media. No GBS detection in vaginal or anorectal samples occurred with Todd-Hewitt enrichment broth.

## Discussion

In our previous study conducted in southern Brazil, the best performance for GBS screening in pregnant women was found when we used an enrichment culture medium that is not commonly used for this purpose (i.e., HPTH medium). However, some concerns arose about the reproducibility of our results in populations that have different lifestyles, live in different geographical locations, different ages, socioeconomic levels, schooling, and gestational ages. To improve GBS screening performance in pregnant women and considering the benefits of this detection protocol, we extended our study of GBS screening with HPTH medium to another geographical region of Parana state in Brazil.

The percentage of pregnant women (28.4%), which were GBS carriers at 35–37 weeks of gestation in the present study, corroborates with study conducted by Feuerschuette et al. [1]. Two other studies reported a lower prevalence: 17.4% [15] and 15.0% [16]. This variation in GBS maternal detection may be attributable to differences in the applied methodologies to detect GBS and characteristics of the studied populations.

The CDC recommends GBS screening in both vaginal and anorectal specimens from pregnant women [9]. The criteria suggested by the CDC were perfectly met in our study, in which the percentage of GBS detection were 23.5% and 21.9% in vaginal and anorectal specimens using the three media, respectively.

Our study corroborates the good HPTH performance that was previously reported in a study that evaluated a population in a medium-sized city [12]. In the previous study, 24.5% of pregnant women were GBS carriage, detected using HPTH medium, Todd-Hewitt enrichment broth, and antibiotic SBA plating. The present study with a larger study sample (n = 496), which was from a different geographic area in the same state (Paraná) in Brazil, the HPTH medium showed better performance in detecting GBS compared to Todd-Hewitt enrichment broth (*p* = 0.0002) (Table 2). The addition of HPTH clearly increased in 10.9% (n = 54) the GBS detection in both specimens. For vaginal samples, the increase was 5.8% (n = 29) and for anorectal, the increase was 5.1% (n = 25).

The addition of Todd-Hewitt enrichment broth presented less improvement in GBS screening in both vaginal and anorectal specimens (n = 9, 1.8%), 1.2% (n = 6) in vaginal, and 0.6% (n = 3) in anorectal (Figs 1 and 2).

None difference was found between HPTH medium and SBA direct plating (p = 0.7579) for GBS screening (Table 2). The detection of GBS in anorectal and vaginal isolated samples by SBA direct plating was 6.7% (n = 33) and 2.4% (n = 12), respectively (Figs 1 and 2). These results clearly demonstrate the applicability of using SBA direct plating, which increases GBS detection mainly in anorectal samples.

The use of HPTH and SBA media together is promising because of their specificities. The HPTH has the ability to recover lower GBS inoculums, and the SBA is associated with faster detection, which is of paramount importance.

Given the reliability of using SBA direct plating, we may conclude that it can identify GBS 24h earlier than HPTH medium and Todd-Hewitt enrichment broth. Expert microbiologists can perform bacterial identification by serology in selected colonies that are suggestive of GBS and release preliminary results within this time. This shorter time for identification can provide faster results once GBS colonized pregnant women could take the newborn babies at risk for early-onset disease.

One difference in our study’s methodology was the use of SBA without antibiotics. Chaves et al. [12] added gentamicin (8 μg/ml) and nalidixic acid (15 μg/ml) to the SBA in an attempt to not compromise GBS detection by the microbiota. This difference in the medium composition, used in our study, resulted in an increase in the detection of GBS in both vaginal and anorectal specimens. One explanation for the findings may be the susceptibility of GBS to the antibiotic that was added to the SBA in the previous study [12].

Similar to the pregnant women at 35–37 group, in the sixty pregnant women at ≥ 38 weeks of gestation group the addition of the HPTH and SBA direct plating increased the sensitivity of GBS screening too. Our results corroborate a study that was conducted in Iran [17], which detected a high percentage of pregnant women with GBS at more than 38 weeks of gestational age.

We conclude that neither HPTH nor SBA direct agar plating can be recommended as a sole medium for GBS screening in pregnant women because both media alone failed to detect GBS in some of the pregnant women. For adequate GBS recovery in culture, the two media should be used together for vaginal and anorectal specimens. Using HPTH medium would be interesting in future studies from different laboratories to detect GBS in pregnant women from different countries.

## Supporting Information

S1 Table Results of cultures, from vaginal and anorectal specimens, in pregnant women at 35–37 weeks of gestation to detect *Streptococcus agalactiae* by Todd-Hewitt enrichment broth (TODD), sheep blood agar (SBA), and Hitchens-Pike-Todd-Hewitt (HPTH) culture media. (PDF)
