# Supplementary material for: Performance of Hitchens-Pike-Todd-Hewitt Medium for Group B Streptococcus Screening in Pregnant Women
Source: PLoS One. 2015 Apr 16;10(4):e0123988. doi: 10.1371/journal.pone.0123988 (PMC4400012; doi:10.1371/journal.pone.0123988)
Supplement: S1 Table — (PDF) [file pone.0123988.s001.pdf]

result	BSAV	BSAAR	HPTHV	HPTHAR
Negative	Negative	Negative	Negative	Negative
Negative	Negative	Negative	Negative	Negative
Negative	Negative	Negative	Negative	Negative
Negative	Negative	Negative	Negative	Negative
Negative	Negative	Negative	Negative	Negative
Positive	Positive	Positive	Negative	Negative
Negative	Negative	Negative	Negative	Negative
Negative	Negative	Negative	Negative	Negative
Negative	Negative	Negative	Negative	Negative
Negative	Negative	Negative	Negative	Negative
Negative	Negative	Negative	Negative	Negative
Negative	Negative	Negative	Negative	Negative
Negative	Negative	Negative	Negative	Negative
Negative	Negative	Negative	Negative	Negative
Positive	Positive	Positive	Negative	Negative
Negative	Negative	Negative	Negative	Negative
Positive	Positive	Positive	Negative	Negative
Negative	Negative	Negative	Negative	Negative
Negative	Negative	Negative	Negative	Negative
Negative	Negative	Negative	Negative	Negative
Negative	Negative	Negative	Negative	Negative
Positive	Positive	Positive	Negative	Negative
Negative	Negative	Negative	Negative	Negative
Negative	Negative	Negative	Negative	Negative
Negative	Negative	Negative	Negative	Negative
Negative	Negative	Negative	Negative	Negative
Negative	Negative	Negative	Negative	Negative
Positive	Negative	Positive	Negative	Negative
Positive	Positive	Negative	Positive	Positive
Negative	Negative	Negative	Negative	Negative
Negative	Negative	Negative	Negative	Negative
Negative	Negative	Negative	Negative	Negative
Positive	Positive	Positive	Positive	Negative
Negative	Negative	Negative	Negative	Negative
Negative	Negative	Negative	Negative	Negative
Negative	Negative	Negative	Negative	Negative
Negative	Negative	Negative	Negative	Negative
Positive	Positive	Positive	Negative	Negative
Positive	Negative	Positive	Positive	Positive
Positive	Negative	Positive	Negative	Negative
Negative	Negative	Negative	Negative	Negative
Positive	Negative	Negative	Negative	Negative
Negative	Negative	Negative	Negative	Negative
Negative	Negative	Negative	Negative	Negative
Negative	Negative	Negative	Negative	Negative
Negative	Negative	Negative	Negative	Negative
Negative	Negative	Negative	Negative	Negative
Negative	Negative	Negative	Negative	Negative
Negative	Negative	Negative	Negative	Negative

Negative	Negative	Negative	Negative	Negative
Negative	Negative	Negative	Negative	Negative
Negative	Negative	Negative	Negative	Negative
Negative	Negative	Negative	Negative	Negative
Negative	Negative	Negative	Negative	Negative
Negative	Negative	Negative	Negative	Negative
Negative	Negative	Negative	Negative	Negative
Negative	Negative	Negative	Negative	Negative
Negative	Negative	Negative	Negative	Negative
Negative	Negative	Negative	Negative	Negative
Negative	Negative	Negative	Negative	Negative
Negative	Negative	Negative	Negative	Negative
Positive	Positive	Positive	Positive	Negative
Negative	Negative	Negative	Negative	Negative
Negative	Negative	Negative	Negative	Negative
Negative	Negative	Negative	Negative	Negative
Negative	Negative	Negative	Negative	Negative
Negative	Negative	Negative	Negative	Negative
Negative	Negative	Negative	Negative	Negative
Positive	Negative	Negative	Positive	Positive
Negative	Negative	Negative	Negative	Negative
Negative	Negative	Negative	Negative	Negative
Positive	Positive	Positive	Negative	Positive
Positive	Positive	Positive	Positive	Positive
Negative	Negative	Negative	Negative	Negative
Negative	Negative	Negative	Negative	Negative
Negative	Negative	Negative	Negative	Negative
Negative	Negative	Negative	Negative	Negative
Negative	Negative	Negative	Negative	Negative
Positive	Positive	Positive	Positive	Positive
Negative	Negative	Negative	Negative	Negative
Negative	Negative	Negative	Negative	Negative
Negative	Negative	Negative	Negative	Negative
Positive	Negative	Negative	Negative	Positive
Negative	Negative	Negative	Negative	Negative
Negative	Negative	Negative	Negative	Negative
Positive	Positive	Positive	Positive	Negative
Positive	Negative	Negative	Positive	Negative
Negative	Negative	Negative	Negative	Negative
Positive	Positive	Negative	Positive	Negative
Positive	Positive	Positive	Negative	Negative
Positive	Positive	Negative	Positive	Positive
Negative	Negative	Negative	Negative	Negative
Negative	Negative	Negative	Negative	Negative
Negative	Negative	Negative	Negative	Negative
Negative	Negative	Negative	Negative	Negative
Negative	Negative	Negative	Negative	Negative
Negative	Negative	Negative	Negative	Negative
Negative	Negative	Negative	Negative	Negative
Negative	Negative	Negative	Negative	Negative

Negative	Negative	Negative	Negative	Negative
Negative	Negative	Negative	Negative	Negative
Negative	Negative	Negative	Negative	Negative
Positive	Positive	Negative	Positive	Positive
Negative	Negative	Negative	Negative	Negative
Positive	Positive	Positive	Positive	Positive
Negative	Negative	Negative	Negative	Negative
Negative	Negative	Negative	Negative	Negative
Negative	Negative	Negative	Negative	Negative
Negative	Negative	Negative	Negative	Negative
Negative	Negative	Negative	Negative	Negative
Positive	Negative	Positive	Positive	Positive
Negative	Negative	Negative	Negative	Negative
Negative	Negative	Negative	Negative	Negative
Negative	Negative	Negative	Negative	Negative
Negative	Negative	Negative	Negative	Negative
Negative	Negative	Negative	Negative	Negative
Negative	Negative	Negative	Negative	Negative
Negative	Negative	Negative	Negative	Negative
Negative	Negative	Negative	Negative	Negative
Negative	Negative	Negative	Negative	Negative
Negative	Negative	Negative	Negative	Negative
Negative	Negative	Negative	Negative	Negative
Positive	Positive	Positive	Positive	Positive
Negative	Negative	Negative	Negative	Negative
Negative	Negative	Negative	Negative	Negative
Negative	Negative	Negative	Negative	Negative
Negative	Negative	Negative	Negative	Negative
Negative	Negative	Negative	Negative	Negative
Negative	Negative	Negative	Negative	Negative
Negative	Negative	Negative	Negative	Negative
Positive	Positive	Negative	Positive	Positive
Negative	Negative	Negative	Negative	Negative
Negative	Negative	Negative	Negative	Negative
Negative	Negative	Negative	Negative	Negative
Negative	Negative	Negative	Negative	Negative
Negative	Negative	Negative	Negative	Negative
Negative	Negative	Negative	Negative	Negative
Positive	Positive	Negative	Positive	Positive
Positive	Positive	Positive	Positive	Positive
Negative	Negative	Negative	Negative	Negative
Negative	Negative	Negative	Negative	Negative
Negative	Negative	Negative	Negative	Negative
Negative	Negative	Negative	Negative	Negative
Negative	Negative	Negative	Negative	Negative
Negative	Negative	Negative	Negative	Negative
Positive	Positive	Positive	Negative	Negative
Negative	Negative	Negative	Negative	Negative
Positive	Negative	Negative	Positive	Positive
Negative	Negative	Negative	Negative	Negative

Negative	Negative	Negative	Negative	Negative
Positive	Positive	Negative	Positive	Negative
Negative	Negative	Negative	Negative	Negative
Negative	Negative	Negative	Negative	Negative
Negative	Negative	Negative	Negative	Negative
Positive	Negative	Negative	Positive	Positive
Negative	Negative	Negative	Negative	Negative
Negative	Negative	Negative	Negative	Negative
Negative	Negative	Negative	Negative	Negative
Negative	Negative	Negative	Negative	Negative
Positive	Positive	Positive	Negative	Negative
Negative	Negative	Negative	Negative	Negative
Negative	Negative	Negative	Negative	Negative
Negative	Negative	Negative	Negative	Negative
Negative	Negative	Negative	Negative	Negative
Positive	Positive	Negative	Positive	Negative
Positive	Positive	Positive	Positive	Positive
Negative	Negative	Negative	Negative	Negative
Positive	Positive	Positive	Negative	Negative
Negative	Negative	Negative	Negative	Negative
Positive	Positive	Positive	Positive	Positive
Negative	Negative	Negative	Negative	Negative
Positive	Negative	Positive	Positive	Positive
Negative	Negative	Negative	Negative	Negative
Negative	Negative	Negative	Negative	Negative
Negative	Negative	Negative	Negative	Negative
Negative	Negative	Negative	Negative	Negative
Negative	Negative	Negative	Negative	Negative
Positive	Negative	Positive	Positive	Positive
Negative	Negative	Negative	Negative	Negative
Negative	Negative	Negative	Negative	Negative
Positive	Negative	Negative	Positive	Positive
Positive	Negative	Negative	Positive	Positive
Negative	Negative	Negative	Negative	Negative
Positive	Negative	Negative	Positive	Positive
Positive	Positive	Negative	Negative	Positive
Positive	Negative	Positive	Positive	Negative
Positive	Negative	Negative	Positive	Negative
Positive	Negative	Positive	Positive	Negative
Negative	Negative	Negative	Negative	Negative
Positive	Negative	Negative	Positive	Positive
Positive	Negative	Negative	Positive	Negative
Negative	Negative	Negative	Negative	Negative
Negative	Negative	Negative	Negative	Negative
Negative	Negative	Negative	Negative	Negative
Positive	Negative	Positive	Negative	Positive
Positive	Positive	Negative	Positive	Positive
Negative	Negative	Negative	Negative	Negative
Positive	Positive	Positive	Positive	Positive
Negative	Negative	Negative	Negative	Negative

Negative	Negative	Negative	Negative	Negative
Negative	Negative	Negative	Negative	Negative
Positive	Positive	Positive	Positive	Positive
Positive	Negative	Positive	Negative	Negative
Negative	Negative	Negative	Negative	Negative
Negative	Negative	Negative	Negative	Negative
Positive	Negative	Negative	Positive	Negative
Positive	Negative	Positive	Positive	Negative
Positive	Negative	Negative	Negative	Positive
Negative	Negative	Negative	Negative	Negative
Positive	Negative	Negative	Positive	Positive
Negative	Negative	Negative	Negative	Negative
Positive	Positive	Negative	Positive	Negative
Negative	Negative	Negative	Negative	Negative
Positive	Positive	Positive	Positive	Positive
Negative	Negative	Negative	Negative	Negative
Negative	Negative	Negative	Negative	Negative
Negative	Negative	Negative	Negative	Negative
Negative	Negative	Negative	Negative	Negative
Positive	Positive	Positive	Positive	Negative
Negative	Negative	Negative	Negative	Negative
Negative	Negative	Negative	Negative	Negative
Negative	Negative	Negative	Negative	Negative
Negative	Negative	Negative	Negative	Negative
Positive	Negative	Negative	Negative	Positive
Positive	Negative	Negative	Negative	Negative
Positive	Positive	Positive	Positive	Negative
Negative	Negative	Negative	Negative	Negative
Negative	Negative	Negative	Negative	Negative
Positive	Negative	Negative	Positive	Negative
Negative	Negative	Negative	Negative	Negative
Negative	Negative	Negative	Negative	Negative
Positive	Negative	Negative	Negative	Positive
Negative	Negative	Negative	Negative	Negative
Negative	Negative	Negative	Negative	Negative
Negative	Negative	Negative	Negative	Negative
Negative	Negative	Negative	Negative	Negative
Negative	Negative	Negative	Negative	Negative
Negative	Negative	Negative	Negative	Negative
Negative	Negative	Negative	Negative	Negative
Negative	Negative	Negative	Negative	Negative
Negative	Negative	Negative	Negative	Negative
Positive	Negative	Positive	Negative	Negative
Positive	Negative	Negative	Positive	Negative
Positive	Negative	Positive	Negative	Positive
Positive	Positive	Negative	Positive	Negative
Negative	Negative	Negative	Negative	Negative
Negative	Negative	Negative	Negative	Negative
Positive	Positive	Positive	Positive	Negative
Negative	Negative	Negative	Negative	Negative

Negative	Negative	Negative	Negative	Negative
Positive	Negative	Negative	Negative	Negative
Negative	Negative	Negative	Negative	Negative
Negative	Negative	Negative	Negative	Negative
Negative	Negative	Negative	Negative	Negative
Positive	Negative	Positive	Positive	Positive
Positive	Negative	Negative	Negative	Positive
Negative	Negative	Negative	Negative	Negative
Negative	Negative	Negative	Negative	Negative
Positive	Positive	Negative	Positive	Positive
Negative	Negative	Negative	Negative	Negative
Negative	Negative	Negative	Negative	Negative
Positive	Positive	Negative	Positive	Negative
Negative	Negative	Negative	Negative	Negative
Positive	Positive	Positive	Positive	Negative
Positive	Positive	Positive	Positive	Negative
Negative	Negative	Negative	Negative	Negative
Negative	Negative	Negative	Negative	Negative
Positive	Positive	Negative	Positive	Negative
Positive	Negative	Positive	Negative	Positive
Negative	Negative	Negative	Negative	Negative
Positive	Negative	Positive	Positive	Positive
Positive	Positive	Positive	Positive	Positive
Positive	Negative	Negative	Positive	Negative
Positive	Negative	Negative	Positive	Positive
Positive	Negative	Negative	Negative	Negative
Positive	Negative	Positive	Positive	Positive
Positive	Negative	Negative	Positive	Negative
Negative	Negative	Negative	Negative	Negative
Positive	Negative	Positive	Positive	Negative
Negative	Negative	Negative	Negative	Negative
Negative	Negative	Negative	Negative	Negative
Negative	Negative	Negative	Negative	Negative
Negative	Negative	Negative	Negative	Negative
Positive	Positive	Positive	Negative	Negative
Negative	Negative	Negative	Negative	Negative
Positive	Positive	Positive	Positive	Positive
Negative	Negative	Negative	Negative	Negative
Negative	Negative	Negative	Negative	Negative
Positive	Negative	Positive	Positive	Positive
Positive	Positive	Negative	Positive	Negative
Negative	Negative	Negative	Negative	Negative
Positive	Positive	Positive	Positive	Positive
Positive	Negative	Negative	Positive	Negative
Positive	Negative	Negative	Negative	Positive
Positive	Negative	Negative	Positive	Negative
Negative	Negative	Negative	Negative	Negative
Positive	Negative	Positive	Negative	Positive
Negative	Negative	Negative	Negative	Negative
Positive	Negative	Positive	Negative	Positive

Negative	Negative	Negative	Negative	Negative
Negative	Negative	Negative	Negative	Negative
Negative	Negative	Negative	Negative	Negative
Negative	Negative	Negative	Negative	Negative
Negative	Negative	Negative	Negative	Negative
Negative	Negative	Negative	Negative	Negative
Negative	Negative	Negative	Negative	Negative
Positive	Positive	Negative	Negative	Negative
Positive	Positive	Positive	Positive	Positive
Negative	Negative	Negative	Negative	Negative
Positive	Positive	Positive	Negative	Negative
Negative	Negative	Negative	Negative	Negative
Negative	Negative	Negative	Negative	Negative
Negative	Negative	Negative	Negative	Negative
Negative	Negative	Negative	Negative	Negative
Negative	Negative	Negative	Negative	Negative
Positive	Negative	Positive	Positive	Positive
Positive	Positive	Positive	Positive	Positive
Positive	Negative	Negative	Negative	Negative
Negative	Negative	Negative	Negative	Negative
Negative	Negative	Negative	Negative	Negative
Positive	Positive	Positive	Negative	Positive
Negative	Negative	Negative	Negative	Negative
Negative	Negative	Negative	Negative	Negative
Negative	Negative	Negative	Negative	Negative
Positive	Positive	Negative	Positive	Negative
Positive	Positive	Negative	Positive	Negative
Positive	Positive	Negative	Positive	Negative
Negative	Negative	Negative	Negative	Negative
Negative	Negative	Negative	Negative	Negative
Positive	Positive	Negative	Negative	Negative
Positive	Positive	Positive	Positive	Positive
Negative	Negative	Negative	Negative	Negative
Positive	Negative	Positive	Negative	Negative
Negative	Negative	Negative	Negative	Negative
Negative	Negative	Negative	Negative	Negative
Positive	Negative	Negative	Negative	Positive
Negative	Negative	Negative	Negative	Negative
Negative	Negative	Negative	Negative	Negative
Positive	Positive	Positive	Positive	Negative
Negative	Negative	Negative	Negative	Negative
Negative	Negative	Negative	Negative	Negative
Negative	Negative	Negative	Negative	Negative
Negative	Negative	Negative	Negative	Negative
Negative	Negative	Negative	Negative	Negative
Negative	Negative	Negative	Negative	Negative
Negative	Negative	Negative	Negative	Negative
Positive	Negative	Negative	Negative	Negative
Negative	Negative	Negative	Negative	Negative
Negative	Negative	Negative	Negative	Negative

Negative	Negative	Negative	Negative	Negative
Negative	Negative	Negative	Negative	Negative
Positive	Negative	Positive	Negative	Positive
Negative	Negative	Negative	Negative	Negative
Negative	Negative	Negative	Negative	Negative
Negative	Negative	Negative	Negative	Negative
Positive	Positive	Positive	Negative	Negative
Negative	Negative	Negative	Negative	Negative
Negative	Negative	Negative	Negative	Negative
Negative	Negative	Negative	Negative	Negative
Negative	Negative	Negative	Negative	Negative
Negative	Negative	Negative	Negative	Negative
Negative	Negative	Negative	Negative	Negative
Negative	Negative	Negative	Negative	Negative
Negative	Negative	Negative	Negative	Negative
Negative	Negative	Negative	Negative	Negative
Negative	Negative	Negative	Negative	Negative
Negative	Negative	Negative	Negative	Negative
Positive	Negative	Negative	Positive	Negative
Positive	Positive	Positive	Negative	Negative
Negative	Negative	Negative	Negative	Negative
Positive	Positive	Positive	Negative	Positive
Positive	Negative	Positive	Negative	Negative
Negative	Negative	Negative	Negative	Negative
Negative	Negative	Negative	Negative	Negative
Negative	Negative	Negative	Negative	Negative
Negative	Negative	Negative	Negative	Negative
Positive	Positive	Positive	Positive	Negative
Negative	Negative	Negative	Negative	Negative
Negative	Negative	Negative	Negative	Negative
Negative	Negative	Negative	Negative	Negative
Negative	Negative	Negative	Negative	Negative
Negative	Negative	Negative	Negative	Negative
Negative	Negative	Negative	Negative	Negative
Negative	Negative	Negative	Negative	Negative
Negative	Negative	Negative	Negative	Negative
Negative	Negative	Negative	Negative	Negative
Negative	Negative	Negative	Negative	Negative
Negative	Negative	Negative	Negative	Negative
Negative	Negative	Negative	Negative	Negative
Positive	Positive	Positive	Positive	Positive
Negative	Negative	Negative	Negative	Negative
Negative	Negative	Negative	Negative	Negative
Positive	Positive	Positive	Positive	Positive
Negative	Negative	Negative	Negative	Negative
Negative	Negative	Negative	Negative	Negative
Negative	Negative	Negative	Negative	Negative
Negative	Negative	Negative	Negative	Negative
Negative	Negative	Negative	Negative	Negative
Positive	Negative	Positive	Positive	Negative

Negative	Negative	Negative	Negative	Negative
Negative	Negative	Negative	Negative	Negative
Negative	Negative	Negative	Negative	Negative
Negative	Negative	Negative	Negative	Negative
Negative	Negative	Negative	Negative	Negative
Positive	Negative	Negative	Positive	Negative
Negative	Negative	Negative	Negative	Negative
Negative	Negative	Negative	Negative	Negative
Negative	Negative	Negative	Negative	Negative
Negative	Negative	Negative	Negative	Negative
Negative	Negative	Negative	Negative	Negative
Negative	Negative	Negative	Negative	Negative
Negative	Negative	Negative	Negative	Negative
Negative	Negative	Negative	Negative	Negative
Negative	Negative	Negative	Negative	Negative
Negative	Negative	Negative	Negative	Negative
Positive	Negative	Positive	Negative	Positive
Negative	Negative	Negative	Negative	Negative
Negative	Negative	Negative	Negative	Negative
Positive	Positive	Positive	Positive	Positive
Positive	Positive	Positive	Positive	Positive
Negative	Negative	Negative	Negative	Negative
Negative	Negative	Negative	Negative	Negative
Negative	Negative	Negative	Negative	Negative
Negative	Negative	Negative	Negative	Negative
Negative	Negative	Negative	Negative	Negative
Negative	Negative	Negative	Negative	Negative
Negative	Negative	Negative	Negative	Negative
Negative	Negative	Negative	Negative	Negative
Negative	Negative	Negative	Negative	Negative
Negative	Negative	Negative	Negative	Negative
Negative	Negative	Negative	Negative	Negative
Negative	Negative	Negative	Negative	Negative
Negative	Negative	Negative	Negative	Negative
Negative	Negative	Negative	Negative	Negative
Positive	Negative	Positive	Positive	Positive
Negative	Negative	Negative	Negative	Negative
Negative	Negative	Negative	Negative	Negative
Negative	Negative	Negative	Negative	Negative
Positive	Positive	Positive	Positive	Positive
Negative	Negative	Negative	Negative	Negative
Negative	Negative	Negative	Negative	Negative
Negative	Negative	Negative	Negative	Negative
Positive	Positive	Negative	Positive	Negative
Negative	Negative	Negative	Negative	Negative
Negative	Negative	Negative	Negative	Negative
Positive	Positive	Positive	Positive	Positive
Negative	Negative	Negative	Negative	Negative
Negative	Negative	Negative	Negative	Negative
Negative	Negative	Negative	Negative	Negative

Negative	Negative	Negative	Negative	Negative
Negative	Negative	Negative	Negative	Negative
Negative	Negative	Negative	Negative	Negative
Positive	Positive	Positive	Positive	Positive
Negative	Negative	Negative	Negative	Negative
Negative	Negative	Negative	Negative	Negative
Negative	Negative	Negative	Negative	Negative
Negative	Negative	Negative	Negative	Negative
Negative	Negative	Negative	Negative	Negative
Negative	Negative	Negative	Negative	Negative
Negative	Negative	Negative	Negative	Negative
Negative	Negative	Negative	Negative	Negative
Negative	Negative	Negative	Negative	Negative
Negative	Negative	Negative	Negative	Negative
Positive	Positive	Negative	Positive	Negative
Negative	Negative	Negative	Negative	Negative
Positive	Positive	Negative	Positive	Positive
Positive	Negative	Negative	Negative	Positive
Negative	Negative	Negative	Negative	Negative
Negative	Negative	Negative	Negative	Negative
Negative	Negative	Negative	Negative	Negative
Negative	Negative	Negative	Negative	Negative
Negative	Negative	Negative	Negative	Negative
Negative	Negative	Negative	Negative	Negative
Negative	Negative	Negative	Negative	Negative
Negative	Negative	Negative	Negative	Negative
Negative	Negative	Negative	Negative	Negative
Negative	Negative	Negative	Negative	Negative
Negative	Negative	Negative	Negative	Negative
Negative	Negative	Negative	Negative	Negative
Positive	Positive	Negative	Negative	Negative
Negative	Negative	Negative	Negative	Negative
Negative	Negative	Negative	Negative	Negative
Negative	Negative	Negative	Negative	Negative
Negative	Negative	Negative	Negative	Negative
Negative	Negative	Negative	Negative	Negative
Negative	Negative	Negative	Negative	Negative
Positive	Negative	Positive	Negative	Negative
Negative	Negative	Negative	Negative	Negative
Positive	Negative	Positive	Negative	Negative
Negative	Negative	Negative	Negative	Negative
Positive	Positive	Negative	Negative	Negative
Positive	Negative	Negative	Positive	Positive
Negative	Negative	Negative	Negative	Negative
Negative	Negative	Negative	Negative	Negative
Negative	Negative	Negative	Negative	Negative
Negative	Negative	Negative	Negative	Negative

TODDV	TODDAR
Negative	Negative
Negative	Negative
Negative	Negative
Negative	Negative
Negative	Negative
Negative	Negative
Negative	Negative
Negative	Negative
Negative	Negative
Negative	Negative
Negative	Negative
Negative	Negative
Negative	Negative
Negative	Negative
Positive	Negative
Negative	Negative
Negative	Negative
Negative	Negative
Negative	Negative
Negative	Negative
Negative	Negative
Positive	Negative
Negative	Negative
Negative	Negative
Negative	Negative
Negative	Negative
Negative	Negative
Negative	Negative
Negative	Negative
Negative	Negative
Negative	Negative
Negative	Negative
Negative	Negative
Negative	Negative
Negative	Negative
Negative	Negative
Negative	Negative
Positive	Negative
Positive	Negative
Negative	Negative
Negative	Negative
Positive	Negative
Negative	Negative
Negative	Negative
Negative	Negative
Negative	Negative
Negative	Negative
Negative	Negative
Negative	Negative

Legend:

Positive - Positive for GBS
Negative - Negative for GBS
BSA - blood sheep agar
V - vaginal
AR - anorectal
GBS - Streptococcus agalactiae
HPTH - Hitchens-Pike-Todd-Hewitt medium
TODD - Todd-Hewitt enrichment broth

Negative	Negative
Negative	Negative
Negative	Negative
Negative	Negative
Negative	Negative
Negative	Negative
Negative	Negative
Negative	Negative
Negative	Negative
Negative	Negative
Negative	Negative
Negative	Negative
Negative	Negative
Negative	Negative
Negative	Negative
Negative	Negative
Negative	Negative
Negative	Negative
Negative	Negative
Positive	Positive
Negative	Negative
Negative	Negative
Negative	Negative
Positive	Negative
Negative	Negative
Negative	Negative
Negative	Negative
Negative	Negative
Negative	Negative
Negative	Negative
Negative	Negative
Negative	Negative
Negative	Negative
Negative	Negative
Negative	Negative
Negative	Negative
Positive	Negative
Positive	Negative
Negative	Negative
Positive	Negative
Negative	Negative
Negative	Negative
Negative	Negative
Negative	Negative
Negative	Negative
Negative	Negative
Negative	Negative
Negative	Negative
Negative	Negative
Negative	Negative

Negative	Negative
Negative	Negative
Negative	Negative
Positive	Negative
Negative	Negative
Negative	Negative
Negative	Negative
Negative	Negative
Negative	Negative
Negative	Negative
Negative	Negative
Negative	Negative
Negative	Negative
Negative	Negative
Negative	Negative
Negative	Negative
Negative	Negative
Negative	Negative
Negative	Negative
Negative	Negative
Negative	Negative
Negative	Negative
Negative	Negative
Negative	Negative
Negative	Negative
Negative	Negative
Negative	Negative
Negative	Negative
Negative	Negative
Negative	Negative
Negative	Negative
Positive	Negative
Negative	Negative
Negative	Negative
Negative	Negative
Negative	Negative
Negative	Negative
Negative	Negative
Positive	Negative
Positive	Negative
Negative	Negative
Negative	Negative
Negative	Negative
Negative	Negative
Negative	Negative
Negative	Negative
Positive	Negative
Negative	Negative
Negative	Negative
Negative	Negative

Negative	Negative
Positive	Negative
Negative	Negative
Negative	Negative
Negative	Negative
Negative	Negative
Negative	Negative
Negative	Negative
Negative	Negative
Negative	Negative
Negative	Negative
Negative	Negative
Negative	Negative
Negative	Negative
Negative	Negative
Negative	Negative
Positive	Negative
Negative	Negative
Negative	Negative
Negative	Negative
Positive	Negative
Negative	Negative
Positive	Positive
Negative	Negative
Negative	Negative
Negative	Negative
Negative	Negative
Negative	Negative
Positive	Negative
Negative	Negative
Negative	Negative
Negative	Negative
Negative	Negative
Negative	Negative
Negative	Negative
Negative	Negative
Negative	Negative
Negative	Negative
Negative	Negative
Negative	Negative
Negative	Negative
Negative	Negative
Negative	Negative
Negative	Negative
Negative	Negative
Negative	Negative
Negative	Negative
Negative	Negative
Negative	Negative
Negative	Negative

Negative	Negative
Negative	Negative
Negative	Negative
Negative	Negative
Negative	Negative
Negative	Negative
Negative	Negative
Positive	Negative
Negative	Negative
Negative	Negative
Negative	Negative
Negative	Negative
Positive	Negative
Negative	Negative
Positive	Positive
Negative	Negative
Negative	Negative
Negative	Negative
Negative	Negative
Negative	Negative
Negative	Negative
Negative	Negative
Negative	Negative
Negative	Negative
Negative	Negative
Positive	Negative
Positive	Negative
Negative	Negative
Negative	Negative
Negative	Negative
Negative	Negative
Negative	Negative
Negative	Negative
Negative	Negative
Negative	Negative
Negative	Negative
Negative	Negative
Negative	Negative
Negative	Negative
Negative	Negative
Negative	Negative
Negative	Negative
Negative	Negative
Negative	Negative
Negative	Negative
Negative	Negative
Negative	Negative
Negative	Negative
Positive	Positive
Negative	Negative

Negative	Negative
Positive	Positive
Negative	Negative
Negative	Negative
Negative	Negative
Positive	Positive
Positive	Negative
Negative	Negative
Negative	Negative
Positive	Negative
Negative	Negative
Negative	Negative
Positive	Negative
Negative	Negative
Positive	Negative
Positive	Negative
Negative	Negative
Negative	Negative
Positive	Positive
Negative	Positive
Negative	Negative
Negative	Negative
Negative	Negative
Negative	Negative
Negative	Negative
Negative	Positive
Negative	Positive
Negative	Negative
Negative	Negative
Negative	Negative
Negative	Negative
Negative	Negative
Negative	Negative
Negative	Negative
Negative	Negative
Negative	Negative
Positive	Negative
Negative	Negative
Negative	Negative
Negative	Negative
Positive	Negative
Negative	Negative
Positive	Negative
Negative	Negative
Negative	Negative
Negative	Negative
Negative	Negative
Negative	Negative
Negative	Negative
Negative	Negative

Negative	Negative
Negative	Negative
Negative	Negative
Negative	Negative
Negative	Negative
Negative	Negative
Negative	Negative
Positive	Negative
Positive	Negative
Negative	Negative
Negative	Positive
Negative	Negative
Negative	Negative
Negative	Negative
Negative	Negative
Negative	Negative
Negative	Negative
Positive	Negative
Positive	Negative
Negative	Negative
Negative	Negative
Negative	Negative
Negative	Negative
Negative	Negative
Negative	Negative
Negative	Negative
Negative	Negative
Positive	Negative
Negative	Negative
Negative	Negative
Positive	Negative
Positive	Negative
Negative	Negative
Negative	Negative
Negative	Negative
Negative	Negative
Negative	Positive
Negative	Negative
Negative	Negative
Positive	Negative
Negative	Negative
Negative	Negative
Negative	Negative
Negative	Negative
Negative	Negative
Negative	Negative
Negative	Negative
Positive	Negative
Negative	Negative
Negative	Negative

Negative	Negative
Negative	Negative
Negative	Negative
Negative	Negative
Negative	Negative
Negative	Negative
Negative	Negative
Negative	Negative
Negative	Negative
Negative	Negative
Negative	Negative
Negative	Negative
Negative	Negative
Negative	Negative
Negative	Negative
Negative	Negative
Negative	Negative
Negative	Negative
Negative	Negative
Negative	Negative
Negative	Negative
Positive	Negative
Negative	Negative
Negative	Negative
Negative	Negative
Negative	Negative
Negative	Negative
Positive	Negative
Negative	Negative
Negative	Negative
Negative	Negative
Negative	Negative
Negative	Negative
Negative	Negative
Negative	Negative
Negative	Negative
Negative	Negative
Negative	Negative
Negative	Negative
Negative	Negative
Positive	Negative
Negative	Negative
Negative	Negative
Negative	Negative
Negative	Negative
Negative	Negative
Negative	Negative
Negative	Negative
Negative	Negative
Negative	Negative

Negative	Negative
Negative	Negative
Negative	Negative
Negative	Negative
Negative	Negative
Negative	Negative
Negative	Negative
Negative	Negative
Negative	Negative
Negative	Negative
Negative	Negative
Negative	Negative
Negative	Negative
Negative	Negative
Negative	Negative
Negative	Negative
Negative	Negative
Negative	Negative
Negative	Negative
Positive	Positive
Negative	Positive
Negative	Negative
Negative	Negative
Negative	Negative
Negative	Negative
Negative	Negative
Negative	Negative
Negative	Negative
Negative	Negative
Negative	Negative
Negative	Negative
Negative	Negative
Negative	Negative
Negative	Negative
Negative	Negative
Negative	Negative
Negative	Negative
Negative	Negative
Negative	Negative
Positive	Negative
Negative	Negative
Negative	Negative
Negative	Negative
Positive	Negative
Negative	Negative
Negative	Negative
Positive	Negative
Negative	Negative
Negative	Negative
Negative	Negative

Negative	Negative
Negative	Negative
Negative	Negative
Negative	Negative
Negative	Negative
Negative	Negative
Negative	Negative
Negative	Negative
Negative	Negative
Negative	Negative
Negative	Negative
Negative	Negative
Negative	Negative
Negative	Negative
Positive	Negative
Negative	Negative
Positive	Negative
Negative	Negative
Negative	Negative
Negative	Negative
Negative	Negative
Negative	Negative
Negative	Negative
Negative	Negative
Negative	Negative
Negative	Negative
Negative	Negative
Negative	Negative
Negative	Negative
Negative	Negative
Positive	Negative
Negative	Negative
Negative	Negative
Negative	Negative
Negative	Negative
Negative	Negative
Negative	Negative
Negative	Positive
Negative	Negative
Negative	Negative
Negative	Negative
Positive	Negative
Positive	Negative
Negative	Negative
Negative	Negative
Negative	Negative
Negative	Negative
